# Conceptualization, measurement and effects of positional issues in the Canadian electoral context

**DOI:** 10.1016/j.heliyon.2019.e01453

**Published:** 2019-04-16

**Authors:** Yannick Dufresne, Catherine Ouellet

**Affiliations:** a Université Laval, Canada; b University of Toronto, Canada

**Keywords:** Political science

## Abstract

It is a well-known fact that the major Canadian political parties now use political marketing tools to segment the electorate and target specific groups of voters. Positional issues are at the centre of this type of micro-targeting strategy. This article demonstrates that positional issues played a greater role in Canadian electoral politics than previously assumed. Despite the many theoretical reasons for why the effects of positional issues might have been overlooked, accounting for disaggregation error shows stable and consistent effect of positional issues on vote choice in Canada. Multi-item issue scales are used to test the stability and relative strength of positional issues compared to rival concepts, such as values and party identification. Once measured through aggregated items, the effects of positional issues on vote choice in Canada might even compare with those of conventional vote predictors, such as party identification. Hence, it shows that the actions parties take to capitalize on positional issues, as described by the political marketing literature, are justified.

## 1 Introduction

There is a persisting idea in Canada that the major Canadian political parties engage in brokerage style politics. In a brokerage politics system, parties avoid to pronouncing themselves on divisive issues that might exacerbate tense social cleavages (Carty et al., 2000; Clarke et al., 1984). As a result, parties end up competing within the same policy space and over the same voters, and few ideological differences are developed between them. The brokerage politics perspective views Canadian parties as “centrist, non-ideological election machines” (Bickerton and Gagnon, 2004, 241). Electoral choice in such a context is influenced largely by leader images and valence issues (Clarke et al., 1996). Valence issues, unlike positional issues, are defined by their non-polarizing nature. They are issues upon which everyone agrees – such as the need for a good economy and clean environment – and do not address the means by which these ends should be achieved. As such, brokerage parties do not fit the rational party model inherent in the classical theory of democracy (Downs, 1957). They do not develop positions on issues in an attempt to target the median voter. Rather, brokerage parties avoid taking any positions on issues at all. Yet classic democratic theory expects citizens to make vote choices based on parties' positions on issues. If parties do not articulate clear issue positions, how can political issues affect the voting behaviour of citizens? The brokerage model implies that positional issues cannot affect voting behaviour in any substantial way. However, Canadian political parties can and do engage in behaviour prescribed by political marketing theory,^1^ to which positional issues are central. Positional issues are at the core of an exchange between citizens who care about a small set of issues and political parties who target these citizens using micro-campaigns. Such behaviour contrasts with the expected behaviour of parties in a brokerage politics system. The main argument of this article is that the potentially impactful role of positional issues in Canadian politics has been overlooked and that measurement through multi-items scales provides new insights on how parties can use positional issues to their electoral advantages. Positional issues not only matter to Canadian voters, but are also fundamental tools in the strategic toolboxes of political parties. And if one is interested in examining the narrow room for electoral manoeuvring that parties might have, one must consider positional issues.

## 2 Theory

### 2.1 Why studying issue voting in Canada ?

The question of the relevance of issues is indeed consequential. If voters had no significant attitudes about positional issues, party platforms and campaign promises would be irrelevant. In fact, why would parties try to reach specific electorate segments with narrow-casted messages if issues do not count ? Canada is a particularly appropriate setting for the investigation of the relevance of issues because it is a tough case: positional issues should not really matter in Canada. The conventional wisdom is that positional issues do not explain electoral behaviour in the country because peculiar political context discourages mainstream political parties from taking strong stands on issues (Clarke et al., 1996). Different reasons have been offered to explain why brokerage politics developed in Canada. One line of reasoning is based on the idea that the country has long been defined by its racial, cultural and regional heterogeneity (see, for instance, Bourassa, 1902; Mallory, 1954; Siegfried, 1907). If parties were to take clear and unambiguous positions on issues, they would risk alienating segments of the highly fragmented Canadian electorate or affecting national unity. Indeed, in Canada, a politics of accommodation originated from the perceived need for “regional brokerage” (Carty et al., 2000, 17), a strategy some consider optimal for winning elections (Clarkson, 2005). Emerging from that line of reasoning is the idea that Canada is particularly susceptible to valence politics, in which policy debates revolve around the ‘who’ and ‘what’ rather than the ‘how’ (Clarke et al., 2010b). Political parties all campaign on the same uncontentious valence issues (Stokes, 1963, 1992). At the same time, rationally uninformed voters minimize their electoral calculations by voting mainly on perceived competence of the parties (Petrocik, 1996) or on incumbent performance and leader evaluations (Fiorina, 1981), thereby exacerbating the irrelevance of positional issues. Some scholars contend that the sociodemographic characteristics and values of Canadians also preclude issues from mattering (Blais et al., 2002). The empirical findings of this article contest the view that voters do not have stable issue preferences to which parties can appeal.

A brokerage politics context and a lack of citizen competence are not the only reasons for why positional issues have been understudied. Positional issues have also been understudied because they are idiosyncratic to each country and thus not always amenable to comparative evaluations. The idiosyncratic nature of positional issues stands in contrast to the ubiquitous nature of valence issues, which are better suited for comparative investigation (see Lewis-Beck, 1988; Powell, Jr. and Whitten, 1993). Positional issues are often heavily dependent on specific “cultural” contexts (Leege et al., 2002). Issues such as race in the United States, Turkish integration in the European Union, the asylum seekers debate in Australia, and the Aboriginal question in Canada are all difficult to examine cross-nationally. The solution might be to move up a level of abstraction by developing concepts with less defined attributes, such as ‘tolerance toward ethnic minorities’. Such an approach can shed light on some research questions, like the global evolution of values in post-industrialized countries (see, for instance, Inglehart, 1977). However, it is difficult to defend the idea that nothing can be gained by being less abstract and developing more precise concepts that retain the contextually-derived symbolic dimensions of issues. The potential impact of issues on the vote cannot be divorced from the way that issues are understood by voters. By not taking into account the unique cultural values inherent to different contexts, we might forego insight into important psychological processes. For instance, without understanding the cultural values of a country, we might not understand why issue framing might heterogeneously affect a demographically similar group of people (Gamson and Modigliani, 1987). Considering the conventional characterization of Canadian electoral dynamics, it is therefore relevant to empirically investigate the possibility that positional issues may have a substantial impact on voting behaviour. Thus, it would show that the actions parties take to capitalize on positional issues, as described by the political marketing literature, are justified (see, for instance, Baines, 1999).

### 2.2 The messy territory of issue conceptualization

The various ways that issues are conceptualized and operationalized in the literature reflects a lack of consensus about their effects on vote choice. The term ‘issue’ is used in the literature to refer to many different concepts and theories. For example, it is difficult to argue that the same ontological understanding of issues is shared by theories of issue ownership, issue evolution, and issue publics. Complicating the situation further are a multitude of issue typologies, of which the positional-valence dichotomy is only one. Of course, different definitions and operationalizations of issues lead to different observed effects on vote choice. The lack of consensus surrounding the definition of issues has led to issues being operationalized and measured in a diversity of ways. As such, it comes as no surprise that the findings regarding the effect of issues on voting behaviour have been mixed. The persistent disagreement in the literature about the actual role of issues in electoral politics calls for further investigation of the topic.

Four aspects of political issues are assessed by the different issue typologies found in the literature. Some scholars focus on the substance or content of issues. For instance, some make the distinction between traditional issues and ‘new’ issues, such as immigration issues, women's issues and environmental issues, all of which emerged as a result of postindustrialization (Inglehart, 1990). Others argue that the symbolic dimensions of certain ‘cultural’ issues can have peculiar effects on voters. In the United States, cultural issues such as nationalism and patriotism, race, gender and religion are all considered to have contributed to the Republican party's electoral success between 1968 and 1988 (Leege et al., 2002). Differing complexity in the substance of issues has also been categorized into typologies. In that vein, Carmines and Stimson (1980) distinguish between ‘easy’ and ‘hard’ issues, with easy issues being those that require little cognitive engagement to grasp and hard issues being those that require comparatively more. Abramowitz (1995) considers abortion, for instance, as an ‘easy issue,’ which impacted the results of the 1992 American presidential election. Baum (2002) defines another class of issues that has the capacity to attract the attention of the politically less interested: ‘soft issues’, such as foreign policy crises, which are easily framed as compelling human dramas (91). Substantive distinctions between issues are particularly relevant to the solution of the aforementioned citizen competence problem. These distinctions add another dimension to the idea that issues cannot matter to politically unaware voters. If issues are grasped by different kinds of voters, their effects on voters might be different and the mechanisms by which they operate might also be different. Moreover, not all issues trigger the same interest in voters. Some scholars evaluate the impact of issues based on their levels of salience among voters. Soroka (2003) distinguishes between ‘prominent’ and ‘sensational’ issues, the former having a concrete impact on people and thus providing little scope for media impact on public opinion, that later having no impact on most people, and thus having a great potential to be media driven. Other authors focus on examining the effects on voters of ‘public issues’ of the day, such as the issue of the Free Trade Agreement during the 1988 Canadian federal election (Johnston, 1992), health care in 2000 (Nadeau et al., 2010), and corruption and accountability in 2006 (Andrew et al., 2006). But a salient issue is not necessarily a public issue and can be different for different groups of voters or ‘issue publics’ (see Krosnick, 1990). Finally, other issue typologies deal with the strategic value of certain issues. These typologies discuss ‘wedge’ issues, for example, which have the potential to influence voters to defect from their parties (Hillygus and Shields, 2008). They also discuss ‘insurgent’ issues (Vavreck, 2009), which divert attention away from ‘unobstrusive’ issues such as the economy (Baum, 2002) and which are more susceptible to agenda-setting. All in all, the conceptualization of issues in the issue-voting literature can easily make the head spin. It is therefore important for anyone that look at issue voting to clearly define which aspect of issues they are looking at. Alternatively, other research challenges the relevance of valence issues. For instance, Evans and Andersen (2004: 32) find that party affiliation often drives issue perceptions in the British context, thus according closely with the claims originally advanced by *The American Voter* (1960).

The issue typology at the centre of this article is the positional-valence issue dichotomy. Valence issues are most often described as public issues, which by definition are consensual among all voters. The effect of valence issues on vote choice is already well demonstrated in the literature (see Clarke et al., 2010a, 2010b). Positional issues, on the other hand, are divisive issues on which citizens disagree. Different measures of positional issues are used in different voting models. Some scholars use the issues that survey respondents consider most important to them personally (see Bélanger and Nadeau, 2009) while others use respondents' attitudes toward public issues of the day, measured with single questions (Blais et al., 2002). Others prescribe the use of multiple measures to better capture issue preferences among mass publics (Ansolabehere et al., 2008; Rose and McAllister, 1990). In fact, by revealing that attitudes on issues are more stable and impactful than previously assumed, Ansolabehere et al. (2008) offer a picture of the American electorate that dramatically contrast with the one portrayed by conventional voting models. In a similar fashion, Rose and McAllister (1990) make the case that issues matter more than previously assumed in British politics. Considering its particular characteristics, Canada should be considered an infertile soil to replicate these last findings. However, this paper shows the opposite: once correctly measured, positional issues can have as strong an effect on vote choice as rival concepts such as party identification and values. Though positional issues might only explain a small percentage of overall electoral change, small percentages can be decisive in determining the winner of an election.

## 3 Hypothesis

The literature on issue effects leads to a central research hypothesis amenable to empirical investigation. The hypothesis examines the possibility that positional issues might have a considerable impact on the voting behaviour of the Canadian electorate, and that this effect has been previously underestimated because of conceptual and disaggregation error.

Hypothesis 1 The effect of issue positions in Canadian elections has been underestimated because issues have been considered one-by-one and not as aggregated dimension-by-dimension.

## 4 Methodology

The data used to explore the hypothesis tested in this article come from the 2004, 2008 and 2011 Canadian Election Study (CES). These three studies contain a large number of issue items that have consistent question wordings. These characteristics allow us to construct consistent and comparable issue scales. Moreover, the 2004 and 2008 CES waves also include a panel composed of respondents who answered the same issue questions in four-year intervals, which allows us to explore the stability of issue attitudes. These elections are both recent and are not considered to be positional-issue oriented (see Gidengil et al., 2012). Looking for effects of positional issues in contexts in which they are less likely to be highly determinant is a good place to start.

The analysis presented in this section uses issue scales composed of multiple measures. Some scholars have already called for an improvement in the quality of measurements used in Canadian voting behaviour research; of these, some have suggested the use of covariance structure analysis (Gidengil, 1992). Ansolabehere et al. (2008) show that there are clear advantages to using issue scales composed of multiple measures instead of individual survey items. The scaling of survey items also effectively manages the problem of measurement error (see Achen, 1975). Nevertheless, it is still possible for nonrandom measurement error to emerge from unidimensional scales (Green and Citrin, 1994). To account for that possibility, the reliability of scales needs to be assessed. The scales used in this analysis are built using the factor scores for the first factor of principal factors factor analyses.

Pearson's correlations and logistic regressions are then used to assess the stability and strength of positional issues. The issue scales are used to compare the effects of positional issues to rival predictors of vote choice. The proper combination of statistical controls is difficult to determine. There is a debate in the political methodology literature between proponents of fully specified models, measured by a high R^2^, and those favouring specific causal inferences by including only control variables that are theoretically prior to the focal independent variables and correlated with both the dependent and independent variables (King, 1991). The choice of proper controls when assessing the effects of positional issues is further complicated by ongoing debates about the exact causal orders of rival concepts such as party identification, values, and positional issues in vote choice models. It is unclear whether these rival concepts need to be included as controls in a statistical model testing for the effect of positional issues on vote choice. To deal with this uncertainty, different models including many different causal orders are tested. Such a procedure can be considered as a partial sensitivity test for the robustness of the results. The next section presents the substance of the issue scales used in the analysis and the results from the test of central hypothesis.

## 5 Results

Eight positional issue scales are used in this analysis. The choice of these issues can be justified in part by their relevance to the political context and in part by the limits imposed by the available questionnaires. The number of issues and items is obviously limited by the CES questionnaire, which was not designed with the precise idea of building issue scales in mind. Consequently, the number of items per scale varies per issue and is not as high as the multi-item scales used in other studies (see Ansolabehere et al., 2008). Despite these limitations, the issue scales in the following analysis are sufficiently robust to test the stability and strength of positional issues. We also test more issues than in previous analyses (see Ansolabehere et al., 2008). Table 1 shows the issues, the direction of their position, and the number of items used to build the different scales. Tables 2, 3, 4, 5, and 6 provide additional issue scales information (see in Appendix).

**Table 1:** Eight positional issues.

Issue	Position	Number of items
Economy	Free market	6
Environment	Environmentalist	2
Foreign/US relations	More involvement, closer ties	5
Law and order	Tough on crime	3
Minority issues	More acceptance	8
Moral issues	Traditional	4
Social programs	Not cut	6
Women issues	Feminist	7

Positional issues are conventionally included as single items in fully specified regression models. The issue with this is that concepts operationalized by single items are prone to disaggregation error. The results of the present analysis show that the stability and the strength of positional issues are greatly enhanced when disaggregation error is taken into account. When multi-item issue scales are used, the effects of positional issues are clearer than when single-item questions are used. These results are important; they provide some support for the conclusions presented by Ansolabehere et al. (2008). They also suggest that results found in the United States apply to the Canadian setting as well. Positional issues appear more stable and their effect on vote choice is significant.

**Fig. 1:**
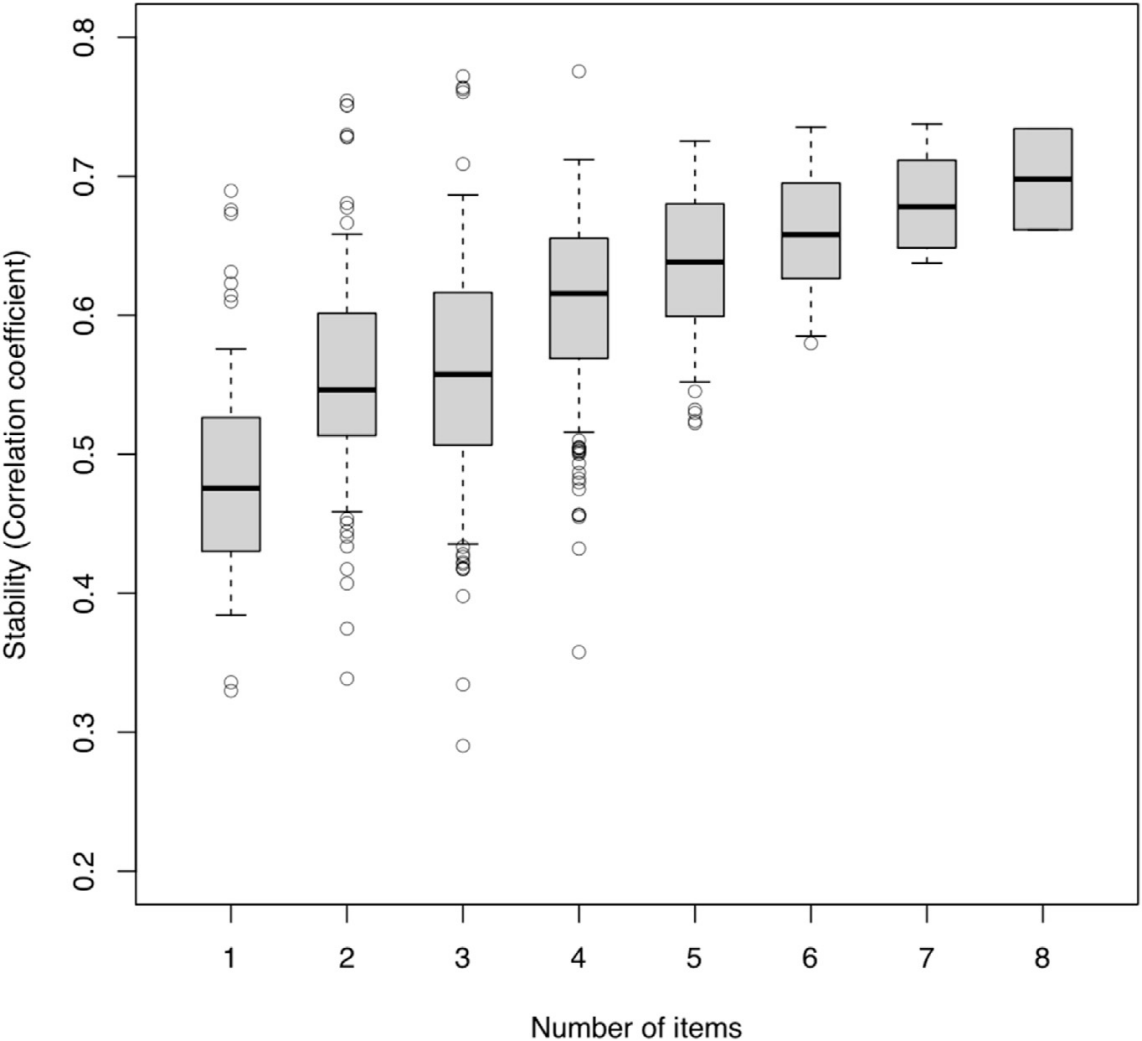
Stability of Issue Attitudes. *Source*: Canadian Election Study (CES), 2004 and 2008 (Panel respondents). *Method*: Pearson’s correlations. Factor scaling (if number of items > 2).

The data reported in Fig. 1 also contribute to the literature in a different way; they illustrate the stability of various issue attitudes between 2004 and 2008. In this case, stability is assessed by examining Pearson's correlations. The higher the score on the vertical axis, the more stable the attitude. These results confirm that aggregating multiple items increases the stability of positional issues. Correlations have been calculated for all possible unique combinations of items for a given issue. For instance, the CES contains seven different measures of attitudes toward the economy, which can be, and were, combined in 127 unique ways for which Pearson's correlations are calculated. Fig. 1 shows the correlations between the 756 unique combinations that can be constructed from the eight positional-issue scales. When it comes to the number of items in the scales, the adage “the more the better” seems to apply (Ansolabehere et al., 2008, 223). Certainly, issue attitudes appear more stable than expected once disaggregation error is taken into account. But this stability does not necessarily mean that positional issues *matter*. And one way to ascertain whether positional issues matter is to examine the effects of the same issues on vote choice.

**Fig. 2:**
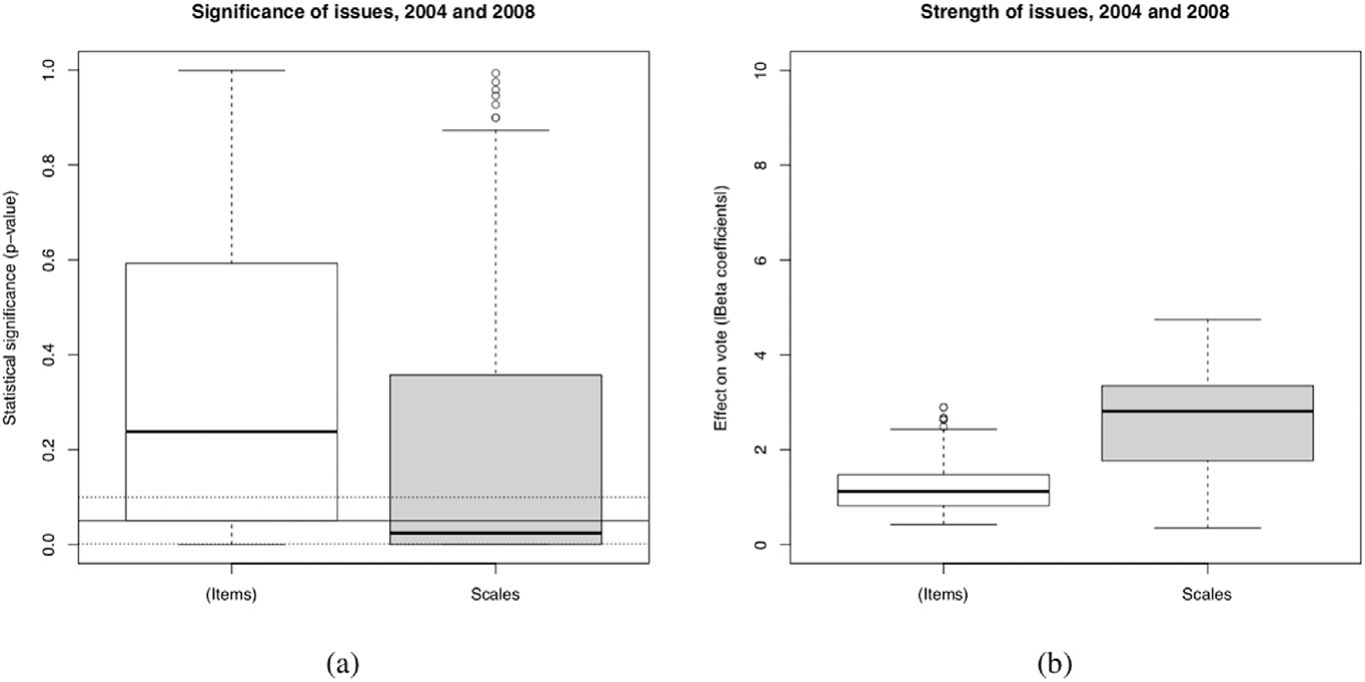
Effect of Issue Items and Scales. *Source*: Canadian Election Study (CES), 2004 and 2008 (Pooled data). *Method*: Logistic regression (summary of 2808 models). Factor scaling. *Dependant variables*: Vote (Conservative, Liberal, NPD). *Independant variables*: *(Items*) (Issue items [included individually]) ; *Scales* (Scales items [included individually]) *Controls:* 1) SES only (468 models); 2) (…) + Values (468 models); 3) (…) + Party identification (468 models) ; 4) (…) + Economic perception (468 models) ; 5) (…) + Leader evaluation (468 models) ; 6) (…) + Incumbent evaluation (468 models). *Note*: The three horizontal lines in graphic (a) represent the statistical significance levels: from bottom to top, *p* < .001; *p* < .05 ; *p*. < .1.

The tests for the effects of issue scales are summarized in Fig. 2. Logistic regression models are estimated to evaluate the statistical significance and the impact of issues on vote choice. Three models, one for each major political party, are estimated for each issue variable. One way to circumvent the methodological debates associated with model specification (see Achen, 2005; Schrodt, 2010) is to incorporate different sets of statistical controls. This procedure can be considered as a partial sensitivity test that assesses the robustness of the findings to the inclusion or exclusion of control variables. In fact, contrary to a sensitivity analysis, control variables were not added individually. The controls were grouped and ordered according to the Michigan school's Funnel of Causality. Fig. 2 (a) reports the statistical significance of the individual issue items and of the issue scales. Fig. 2 (b) shows the strength of the effect on vote choice for the same variables. Once again, the key finding is that scales clearly perform better than individual items when assessing the effects of positional issues on vote choice. And positional issues do have significant effects on vote choice. These results are consistent with those found by Ansolabehere et al. (2008) in the United States.^2^

**Fig. 3:**
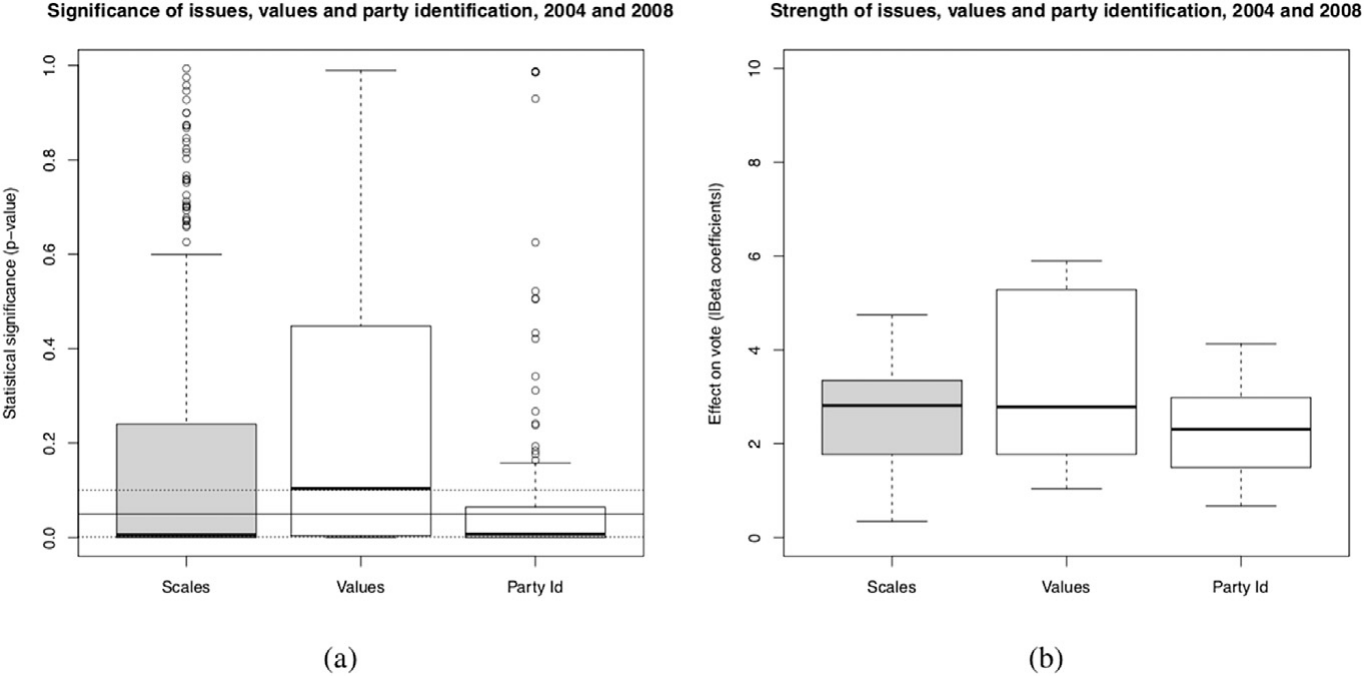
Effect of Issues, Values and Partisan Identifications. *Source*: Canadian Election Study (CES), 2004 and 2008. *Method:* Binary logistic regression (summary of 2808 models.) Factor scaling. *Dependant variables*: Vote (Conservative), Vote (Liberal), Vote (NDP). *Independant variables: Scales* (Issue scales [included individually]) ; *Values* (value scales [included individually]) ; *Party Id* (Partisan identification [included individually]) *Controls*: 1) SES only (468 models) ; 2) (…) + Values (468 models); 3) (…) + Party identification (468 models) ; 4) (…) + Economic perception (468 models) ; 5) (…) + Leader evaluation (468 models); 6) (…) + Incumbent evalution (468 models). *Note:* The three horizontal lines in graphic (a) represent the statistical significance levels: from bottom to top, *p* < .001; *p* < .05; *p* < .1.

How do these effects compare with other predictors of vote choice? Fig. 3 reproduces the results for the issue scales and compares them to the statistical significance and strength of different values and partisan identifications.

**Fig. 4:**
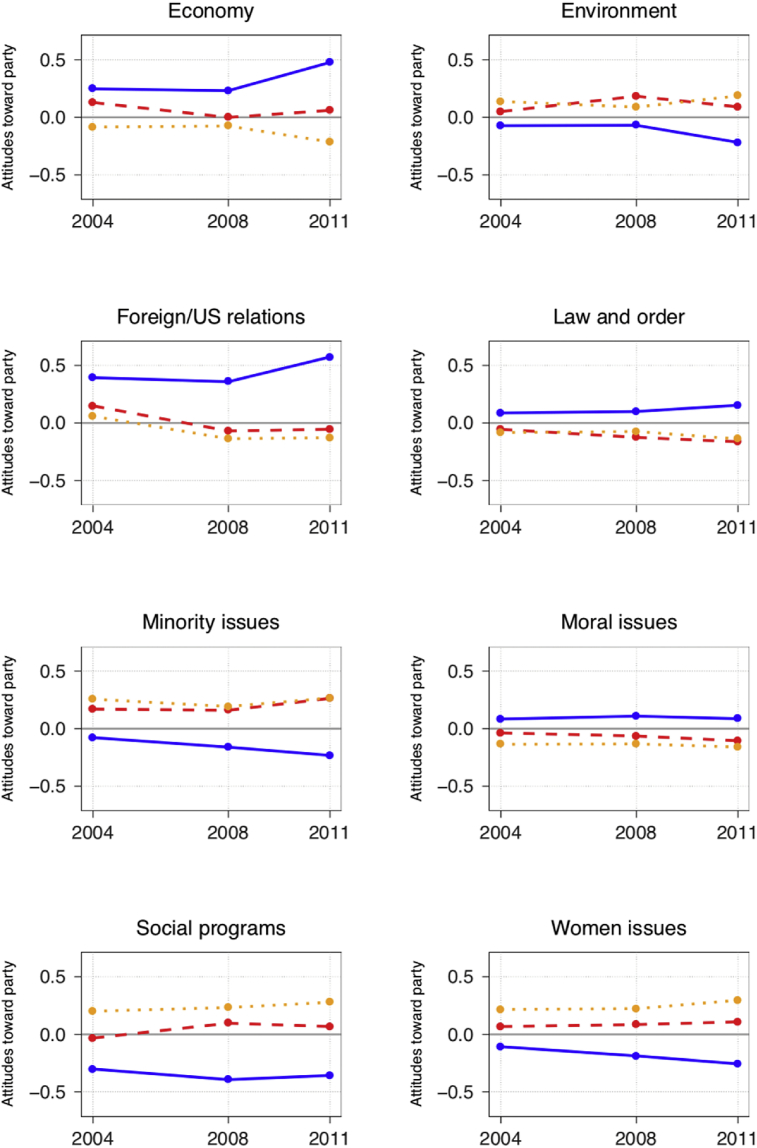
Evolution of Issue Effects for Each Party. *Source*: Canadian Election Study (CES), 2004, 2008 and 2011. *Method*: Linear regression models are used in order to facilitate the direct comparisons between coefficients from different years. *Note*: Solid line = Conservatives; Dashed line = Liberals; Dotted line = New Democrats.

But is it possible that these results just reflect peculiarities of the 2004 and 2008 elections? And is it possible that there has been a recent increase in issue voting? To test these possibilities, the impacts of the eight issue scales are observed over time. The Fig. 4 shows the evolution of the effect for each positional issue from 2004 to 2011. Unfortunately, the cross-time variability of the available CES issue questions makes it possible to only develop comparable issue scales for three elections. The findings presented do not appear to support the idea that there has been a general increase of issue effects. The impact of issue effects seems to vary by election, by party and by positional issue. And in directions that make sense. For example, it is not surprising that the economic positional issue had a significantly more powerful impact in 2011 than in other years, given that the world was facing a major economic crisis at the time. Even so, the effect of economic positions is only greater in 2011 than in 2004 for attitudes toward the most right-wing party (Conservative Party) and the most left-wing party (NDP). But the data do underscore a significant point; it seems that more specific contextual factors also matter. For instance, the effect of the environmental issue significantly increased from 2004 to 2008 for the Liberals, but not for the other parties. It is possible that the unprecedented emphasis that the Liberals put on imposing a carbon tax in 2008 contributed to this situation, especially given that the effect of the environment issue receded after the 2008 Federal Election. Overall, the directions of the issue effects do generally seem to reflect the policy positions adopted by the three parties. It is perhaps interesting to note, however, that every positional issue has its strongest effect on attitudes toward the Conservative Party. Whether such a situation is the result of the Conservatives' global positioning or their electioneering style remains an open question.

## 6 Discussion

The preceding analysis challenges the enduring view that positional issues do not matter much in elections, especially in the Canadian context. The results show that conceptualization and measurement of positional issues through multi-items scales lead to the exposure of both their stability and their significant effect on vote choice. The significance and strength of issues is found to be comparable to even the significance and strength of more conventional predictors of vote choice like values and partisan identification. In fact, it turns out that issues are more often statistically significant than values. However, the conceptual confusion between values and positional issues might explain this surprising observation. One might argue that issue scales are actually measuring values. After all, moral and economic issue scales are often built using almost the same items as moral traditionalism and market liberalism value scales (see, for instance, Gidengil et al., 2012). There is some support in the literature for the proposition that these scales are measures of values (McClosky and Zaller, 1984). But there is also support for considering these scales as measures of issue positions (Ansolabehere et al., 2008). These findings add some nuance to the theoretical frameworks inherited from the Columbia and Michigan schools, which hold that the effect of positional issues on vote choice is negligible. However, these results support the political marketing perspective, that assumes that voter preferences and party strategies have an impact on electoral outcomes (O'Shaughnessy, 1990; Scammell, 1999). And because different positional issues do matter and affect different segments of the population, political parties have good reasons to engage in tactical actions – such as direct mailing or wedge politics – prescribed by political marketing theory.

It is possible that the analysis showing that positional issue attitudes are stable and have independent effects on vote choice is simply the product of the use of multi-item scales. The observed strength of issues could be an artifact of applying the scaling technique typically used to measure values to positional issues. But even so, the idea that scaling techniques determine the strength of issues has little theoretical consequence for the causal order of conventional voting behaviour models. It is discussed above that single-item issue indicators are highly prone to disaggregation error and are thus likely to produce noisy attitudinal signals. These signals become clearer with proper scaling. That noise does not mean that positional issue attitudes are less genuine. Many scholars after Converse (1964) defended the idea that citizens do have political attitudes despite their apparent lack of political sophistication and ideological consistency (Achen, 1975; Lane, 1962; Saris and Sniderman, 2004). Issue scales such as those used in this analysis, as well as those used by Ansolabehere et al. (2008), seem to relax the requirements for political sophistication enough to render citizens able to vote on positional issues. The more items in a scale, the more opportunities to capture citizens' real attitudes. The implication of that line of speculation is that scales might need to include more items in order to correctly measure positional issues. Including several questions about gay rights in a scale, for instance, might help to ensure that it is indeed attitudes about gay rights that are being measured and not attitudes about other, related moral issues, such as abortion. The same pattern should emerge if there are more items available in a scale to measure attitudes toward health care independently from attitudes toward other social programs.

There are many aspects of issue effects that require further investigation. But the principle question at hand is: do different issues affect all voters equally? Different ways of assessing the effects of issues on voters vary both in terms of measurement techniques and conceptualization. The positions and the personal saliences of issues constitute two different dimensions, which need to be measured separately (Miller and Peterson, 2004). These two aspects—position and salience—refer to two of the three elements that limit the potential effect of issues, according to the authors of *The American Voter* (1960).

Electoral studies only have to gain from paying more attention to the conceptualization, measurement, and effect of positional issues. Recent advancements in the field, especially those regarding wedge politics—are frequently at odds with the theoretical assumptions underling the Columbia and Michigan models. In addition to better measurement, voting theories might gain from trading some parsimony for the better integration of the complexities involved in issue voting, such as those pertaining to contextual and heterogeneous effects. After all, positional issues are an essential tool in the democratic exchange between political parties and citizens.

## Declarations

### Author contribution statement

Y. Dufresne: Conceived and designed the experiments; Performed the experiments; Analyzed and interpreted the data; Contributed reagents, materials, analysis tools or data; Wrote the paper.

C. Ouellet: Analyzed and interpreted the data; Contributed reagents, materials, analysis tools or data; Wrote the paper.

### Funding statement

This research did not receive any specific grant from funding agencies in the public, commercial, or not-for-profit sectors.

### Competing interest statement

The authors declare no conflict of interest.

### Additional information

No additional information is available for this paper.

## Footnotes

1 Political marketing parallels the *Marketing Revolution* that occurred in the American business community in the 1950s (see Keith, 1960). Thus, to have any definitional value, political marketing must refer to the application of a consumer-centric concept to the political realm (O'Shaughnessy, 1990). Lilleker and Lees-Marshment (2005) suggest that “as an activity, political marketing is about political organisations (such as political parties, parliaments and government departments) adapting techniques (such as market research and product design) and concepts (such as the desire to satisfy voter demands) originally used in the business world to help them achieve their goals (such as win elections or pass legislation)” (7).

2 Note that Ansolabehere et al. (2008) results are only based on two issue scales: Economy (13 items) and Moral (6 items). The authors also tried “adding a Foreign Policy Scale but it was never statistically or substantively significant in 1992 or 1996” (Ansolabehere et al, 2008: 226). Contrary to these results, all eight positional issue scales are statistically or substantively significant in Canada in at least one of the elections under study. For a comparison of effect of scales on the explained variance, see Table 7 in Appendix.
