# Supplementary material for: Conceptualization, measurement and effects of positional issues in the Canadian electoral context
Source: Heliyon. 2019 Apr 16;5(4):e01453. doi: 10.1016/j.heliyon.2019.e01453 (PMC6475873; doi:10.1016/j.heliyon.2019.e01453)
Supplement: Appendix [file mmc1.docx]

**APPENDIX**

Table 2 : Issue scales information (1)

| Issue scales | Issue items | Factor loadings |
| --- | --- | --- |
| Economy  (Position: Free market) | When businesses make a lot of money, everyone benefits, including the poor. | 0.69 (2004)  0.62 (2008) |
| a = 0.59 (2004) ; 0.62 (2008) | How much confidence have in: Big business. | 0.66 [2004)  0.58 (2008) |
| *Eigenvalues* = 2.03 (2004) ;  2.11 (2008) | Overall, free trade with the U.S. has been good for the Canadian economy.  International trade creates more jobs in Canada than it destroys.  And corporate taxes : should corporate taxes be increased, decreased or kept about the same ?  If people can’t find work in the region where they live, they should move to where the jobs are. | 0.64 (2004)  0.69 (2008)  0.58 (2004)  0.55 (2008)  0.53 (2004)  0.61 (2008)  0.31 (2004)  0.50 (2008) |
| Environment  (Position: Environmentalist) | ... the Environment ? Should the Federal government  spend more, less, or about the same as now ? | 0.79 (2004)  0.79 (2008) |
| a = 0.40 (2004) ; 0.41 (2008) | Protecting the environment is more important than  creating jobs. | 0.79 (2004)  0.79 (2008) |
| *Eigenvalues* = 1.25 (2004) ;  1.26 (2008) |  |  |

*Source*: Canadian Election Study (CES), 2004 and 2008. *Note :* Eigenvalues for the first factors.

Table 3 : Issue scales information (2)

| Issue scales | Issue items | Factor loadings |
| --- | --- | --- |
| Foreign/US relations  (Position: More involvement, closer ties) | How do you feel about the United States ? | 0.71 (2004)  0.63 (2008) |
| a = 0.54 (2004) ; 0.51 (2008) | Do you think Canada’s ties with the United States should be much closer, somewhat closer, about the same as now, somewhat more, distant or much more distant ? | 0.64 [2004)  0.71 (2008) |
| *Eigenvalues* = 1.74 (2004) ;  1.76 (2008) | …Defence ? [Or….Military] spending ? Should the Federal government spend more, less, or about the same as now ?  How much confidence have in : The armed forces.  Canada should participate in peacekeeping operations abroad even if it means putting the lives of Canadian soldiers at risk. | 0.64 (2004)  0.60 (2008)  0.46 (2004)  0.72 (2008)  0.45 (2004)  0.35 (2008) |
| Law and order  (Position: Tough on crime)  a = 0.61 (2004) ; 0.63 (2008) | Do you favor or oppose the death penalty for people convicted of murder ? | 0.75 (2004)  0.72 (2008) |
| *Eigenvalues* = 1.68 (2004) ;  1.70 (2008) | What is the best way to deal with young offenders who commit violent crime : One, give them tougher sentences ; or Two, spend more on rehabilitating them ?    We must crack down on crime, even if that means that criminal lose their rights. | 0.74 (2004)  0.78 (2008)  0.74 (2004)  0.76 (2008) |

*Source*: Canadian Election Study (CES), 2004 and 2008. *Note :* Eigenvalues for the first factors.

Table 4 : Issue scales information (3)

| Issue scales | Issue items | Factor loadings |
| --- | --- | --- |
| Minority issues  (Position: More acceptance) | Immigrants make an important contribution to this country. | 0.70 (2004)  0.67 (2008) |
| a = 0.76 (2004) ; 0.76 (2008) | Too many recent immigrants just don’t want to fit into Canadian society. [Reversed]  We should look after Canadians born in this country first and others second. [Reversed] | 0.67 [2004)  0.69 (2008)  0.66 (2004)  0.69 (2008) |
| *Eigenvalues* = 3.00 (2004) ;  2.95 (2008) | … and racial minorities ? Use any number from zero to one hundred.  Do you think Canada should admit : more immigrants, fewer immigrants, or about the same as now ?  Which statement comes closest to your own view : 1) If Aboriginal peoples tried harder, they could be as well off as other Canadians ; 2) Social and economic conditions make it almost impossible for most Aboriginal peoples to overcome poverty ; 3) Not sure.  How do you feel about aboriginal people ?  It is more difficult for non-whites to be succcessful in  Canadian society than it is for whites. | 0.64 (2004)  0.62 (2008)  0.62 (2004)  0.63 (2008)    0.58 (2004)  0.51 (2008)    0.54 (2004)  0.52 (2008)  0.44 (2004)  0.49 (2008) |

*Source*: Canadian Election Study (CES), 2004 and 2008. *Note :* Eigenvalues for the first factors.

Table 5 : Issue scales information (4)

| Issue scales | Issue items | Factor loadings |
| --- | --- | --- |
| Social programs  (Position: No cut) | The welfare state makes people less willing to look after themselves. [Reversed] | 0.62 (2004)  0.65 (2008) |
| a = 0.56 (2004) ; 0.57 (2008) | … Welfare ? Should the Federal government spend more, less, or about the same as now ? | 0.61 (2004)  0.65 (2008) |
| *Eigenvalues* = 1.96 (2004) ;  1.98 (2008) | … Health Care ? Should the Federal government spend more, less, or about the same as now ?  How much do you think should be done to reduce the gap between the rich and the poor in Canada : much more, somewhat more, about the same as now, somewhat less, or much less ?    … Education ? Should the Federal government spend more, less, or about the same as now ?  Do you favor or oppose having some private hospitals in Canada ? | 0.60 (2004)  0.47 (2008)  0.57 (2004)  0.64 (2008)  0.55 (2004)  0.48 (2008)    0.48 (2004)  0.51 (2008) |

*Source*: Canadian Election Study (CES), 2004 and 2008. *Note :* Eigenvalues for the first factors.

Table 6 : Issue scales information (5)

| Issue scales | Issue items | Factor loadings |
| --- | --- | --- |
| Moral Issues  (Position: Traditional) | Do you favor or oppose same-sex marriage,  or do you have no opinion on this ? | 0.87 (2004)  0.88 (2008) |
| a = 0.80 (2004) ; 0.80 (2008) | Gays and lesbians should be allowed to get  married. [Reversed] | 0.89 [2004)  0.91 (2008) |
| *Eigenvalues* = 2.57 (2004) ;  2.55 (2008) | ...And gays and lesbians ? Use any number from  zero to one hundred.  Do you think it should be: very easy for women to get  an abortion, quite easy, quite difficult, or very difficult ? | 0.83 (2004)  0.79 (2008)  0.57 (2004)  0.57 (2008) |
| Women’s issues  (Position: Feminist) | The feminist movement : 1) Just tries to get equal treatment for women ; 2) Puts men down | 0.71 (2004)  0.73 (2008) |
| a = 0.62 (2004) ; 0.63 (2008) | The feminist movement encourages women : 1) To be independant and speak up for themselves ; 2) To be selfish and think only of themselves. | 0.71 (2004)  0.72 (2008) |
| *Eigenvalues* = 2.27 (2004) ;  2.26 (2008) | How much do you think should be done for women : much more, somewhat more, about the same as now, somewhat less, or much less ?  The best way to protect women’s interests is to have more women in Parliament  … and feminists ? Use any number from zero to one hundred.  Discrimination makes it extremely difficult for women to get jobs equal to their abilities.  Society would be better off if more women stayed home with their children [Reversed] | 0.56 (2004)  0.51 (2008)  0.55 (2004)  0.53 (2008)  0.54 (2004)  0.55 (2008)  0.53 (2004)  0.51 (2008)  0.30 (2004)  0.33 (2008) |

*Source*: Canadian Election Study (CES), 2004 and 2008. *Note :* Eigenvalues for the first factors.

Table 7 : United States vs. Canada : The effect of scales on explained variance

|  | ANES 1992 | ANES 1996 | | CES 2004 | CES 2008 | CES 2011 |
| --- | --- | --- | --- | --- | --- | --- |
|  |  |  | |  |  |  |
| Baseline model (Pseudo-R^2^) | 0.570 | 0.590 | 0.386 | | 0.394 | 0.465 |
| Scales (2) * | + 0.090 | + 0.070 | +0.013 | | +0.004 | +0.004 |
| Scales (8) * | --- | --- | +0.050 | | +0.077 | +0.051 |

*Source :* American National Election Study (ANES), 1992 and 1996. Canadian Election Study (CES), 2004-2011.

*Method*: Probit regression (Adjusted Pseudo-R^2^)

** Note*: The “baseline” models include only Party ID and ideology scales as independant variables.

* *Note*: Ansolabehere et *al.* (2008) results are only based on two issue scales : Economy (13 items) and Moral (6 items). The authors also tried “adding a Foreign Policy Scale but it was never statistically or substantively significant in 1992 or 1996” (Ansolabehere et *al.*, 2008 : 226). Contrary to these results, all eight positional issue scales are statistically or substantively significant in Canada in at least one of the elections under study.
